# Methods for selecting fixed-effect models for heterogeneous codon evolution, with comments on their application to gene and genome data

**DOI:** 10.1186/1471-2148-7-S1-S5

**Published:** 2007-02-08

**Authors:** Le Bao, Hong Gu, Katherine A Dunn, Joseph P Bielawski

**Affiliations:** 1 Department of Mathematics and Statistics, Dalhousie University Halifax Nova Scotia, Canada; 2 Department of Biology, Dalhousie University, Halifax Nova Scotia, Canada

## Abstract

**Background:**

Models of codon evolution have proven useful for investigating the strength and direction of natural selection. In some cases, *a priori *biological knowledge has been used successfully to model heterogeneous evolutionary dynamics among codon sites. These are called fixed-effect models, and they require that all codon sites are assigned to one of several partitions which are permitted to have independent parameters for selection pressure, evolutionary rate, transition to transversion ratio or codon frequencies. For single gene analysis, partitions might be defined according to protein tertiary structure, and for multiple gene analysis partitions might be defined according to a gene's functional category. Given a set of related fixed-effect models, the task of selecting the model that best fits the data is not trivial.

**Results:**

In this study, we implement a set of fixed-effect codon models which allow for different levels of heterogeneity among partitions in the substitution process. We describe strategies for selecting among these models by a backward elimination procedure, Akaike information criterion (AIC) or a corrected Akaike information criterion (AICc). We evaluate the performance of these model selection methods via a simulation study, and make several recommendations for real data analysis. Our simulation study indicates that the backward elimination procedure can provide a reliable method for model selection in this setting. We also demonstrate the utility of these models by application to a single-gene dataset partitioned according to tertiary structure (abalone sperm lysin), and a multi-gene dataset partitioned according to the functional category of the gene (flagellar-related proteins of *Listeria*).

**Conclusion:**

Fixed-effect models have advantages and disadvantages. Fixed-effect models are desirable when data partitions are known to exhibit significant heterogeneity or when a statistical test of such heterogeneity is desired. They have the disadvantage of requiring *a priori *knowledge for partitioning sites. We recommend: (i) selection of models by using backward elimination rather than AIC or AICc, (ii) use a stringent cut-off, *e.g.*, *p *= 0.0001, and (iii) conduct sensitivity analysis of results. With thoughtful application, fixed-effect codon models should provide a useful tool for large scale multi-gene analyses.

## Background

The ratio *d*_N_/*d*_S _(*ω*) has proven a valuable index of the strength and direction of selection pressure. Because genetic data are typically subject to a diversity of evolutionary constraints, estimating *ω *as an average over many sites diminishes the effectiveness of this approach [1]. Statistical power is substantially improved, however, by accommodating variable selection pressures among sites (*e.g*., [2-4]). We follow Kosakovsky Pond and Frost [5] by placing such methods in three groups: (i) the counting methods, which estimate *ω *from counts of substitutions at individual sites (*e.g*., [3]), (ii) the random-effect models, which assume a parametric distribution of variability in the *ω *ratio across sites (*e.g*., [2]), and (iii) the fixed-effect models, which assume sites can be assigned *a priori *to different partitions [4]. The most generalized form of the fixed-effect models treats each site as an independent partition [5,6].

The recent growth of genome scale sequencing projects has sparked interest in using codon models to study mechanisms of innovation and functional divergence in genome-scale datasets [7]. Although the fixed-effect models were developed for analysis of multiple partitions of sites within a single gene, they are also appropriate for joint analyses of multi-gene datasets [4,8]. Fixed-effect models categorize codon sites into different classes which are allowed to have heterogeneous evolutionary dynamics, and such partitions are easily delineated on the basis of complete gene sequences. Moreover, by partitioning genes according to criteria such as their functional category, or role in a metabolic pathway, the fixed-effect models provide a statistical framework for making use of such information when analysing multi-gene datasets.

Yang and Swanson [4] introduced six fixed-effect models (Table 1) based on the codon model of Goldman and Yang [9]. The simplest model (A) assumes that the pattern of substitution is homogeneous over all sites; *i.e*., there are no partitions under model A. Branch lengths are included as parameters of the model. The most complex model (F) treats the different site partitions as independent datasets, having independent model parameters. As it involves a substantial increase in branch length parameters, model F is not recommended for datasets with many partitions [4]. The remaining four models (B-E in Table 1) lie between A and F in complexity. These four models scale the branch lengths of *k *partitions according to the parameter *c*_*k*_, which is a multiple of the branch lengths of the first partition; hence *c*_1 _= 1. Models B through E differ in their treatment of parameters *ω*, *κ *(transition to transversion ratio) and *π *(codon frequencies) among partitions (Table 1). We implemented 11 more fixed-effect models, which represent all the remaining combinations of heterogeneity or homogeneity among partitions for the parameters *c*, *ω*, *κ *and *π *(Table 2). A full description of the fixed effect models and the details of our implementation are presented in the methods section. Hereafter we refer to the complete set of fixed-effect (FE) models by using the revised notation shown in table 2 (FE1–FE16). Note that a capacity to specify fixed-effects under the alternative formulation of Muse and Gaut [10] is available through the program HyPhy [11], although it has not been documented.

**Figure 1:**
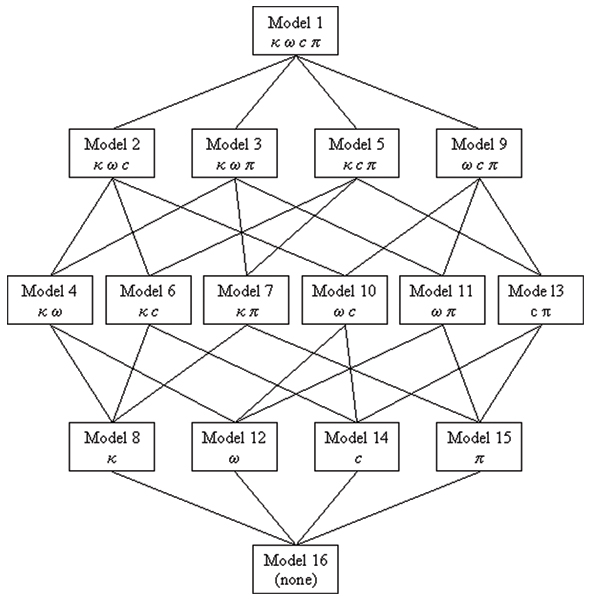
**Relationships among fixed-effect codon models**. The most complex model (FE1) is located at the top and a completely homogenous model (FE16) is located at the bottom. Parameters heterogeneous among partitions for a given model are shown after the model name. Lines between models indicate "1-step" differences in complexity among the models.

Given a related set of fixed-effect models (Figure 1), one is immediately faced with the non-trivial task of selecting the model that best fits the data in hand. Likelihood ratio tests (LRTs) have been shown to be a powerful and reliable means of testing site specific heterogeneity in selective pressure [8,12]. However, Figure 1 illustrates that there are 32 possible nested comparisons of models. It is not desirable to conduct 32 LRTs because computational costs are expensive for datasets with too many sequences or partitions. A popular method of model selection based on LRTs is "backward elimination" [13-15]. Backward elimination reduces a comparatively complex model to a simpler one in a step-by-step fashion. An alternative to "backward elimination" is the Akaike Information Criterion (AIC) [16], where the model with the smallest AIC score is chosen as the ideal model. For a small sample correction, typically when the number of observations is less than 40 times the number of parameters in the model [17], we borrowed the corrected Akaike Information Criterion (AICc) originally developed by Hurvich and Tsai [17] for regression settings.

Although the statistical issues surrounding model selection are well known within the field of molecular evolution [18-20], the established statistical techniques have not been applied to the fixed-effect setting. In this study we used computer simulation to evaluate the performance of backward elimination, AIC and AICc for selecting an optimal model from an array of models specifying different levels of heterogeneity among partitions. We then illustrated the application of these methods on two real datasets. The first was comprised of the buried and exposed sites of the abalone sperm lysin gene; this lysin partition was one of the original test cases of Yang and Swanson [4]. For the second case, we examined the evolutionary heterogeneity of a multi-gene dataset; the region of the genome encoding all the components of the flagellar system of *Listeria *species and several proteins of unknown function.

## Results

### Simulated data

A simulation study was used to measure the accuracy of fixed-effect model selection. We simulated under the 16 different scenarios for heterogeneous codon evolution among data partitions shown in table 2 (see methods for a detailed description of the simulation study), and measured the number of cases where each procedure identified the correct generating model. The backward elimination procedure uses the likelihood ratio test (LRT) to simplify a complex model one parameter at a time; in this case we start at the top of Figure 1 (FE1) and use the LRT to remove non-significant parameters in a step-wise fashion. A more detailed description is provided in the methods. When we applied the LRT under a cut-off probability of 0.05, the backward elimination procedure provided more accurate model specification than either AIC or AICc in all the cases except for model 2 (Table 3). Among all 336 datasets, the accuracy of backward elimination was 78% whereas the accuracy of AIC and AICc was 63% and 64% respectively (Table 3). Note that each model can be related to all other models by the number of connections, or "steps", between them in Figure 1. For all models that are wrongly specified by backward elimination, most were just one step away from the true model (85%). Among these 1-step wrong models, there was a bias in the direction of greater complexity for one of *ω*, *κ *or *c*; replicates heterogeneous for these parameters were never misclassified as homogenous. Taken over all replicates homogenous for *ω*, *κ *or *c*, this bias was generally low, with 1-step error rates of 13%, 9% and 9% respectively.

Similar results were observed for AIC and AICc. Most misclassifications were 1-step errors (77% and 78%), with a bias in the direction of greater complexity for parameter *ω*, *κ *or *c*. Again, heterogeneous replicates were not misclassified as homogenous for these parameters. The 1-step error rates across replicates homogenous for *ω*, *κ *or *c *were 28%, 18% and 26% for AIC, and 26%, 17% and 22% for AICc.

As the number of misclassification errors ≥2-steps was much smaller than the number of 1-step errors, we examined these as an average over backward elimination, AIC and AICc. In 90% of the cases these errors resulted in too simple a model. The involved parameters were *ω*, *c *and *π*; the *κ *parameter was rarely misclassified.

We simulated under two models of codon frequencies: (i) unbiased (*π*_*i *_= 1/61) and (ii) biased frequencies taken from empirical frequencies of the lysin gene. In composite datasets with a 90:10 partition the number of codons in the smaller partition is too low for reliable empirical estimation of 61 different codon frequencies ("F61" method). Hence, in only those cases we used the "F3 × 4" method, which computes codon frequencies from nucleotide frequencies at the three positions of the codon [9]. In the 50:50 and 70:30 datasets we used the empirical estimates of codon frequencies (F61) in each partition. We note that such empirical estimates do not satisfy the requirements of LRT [21] and, hence, the backward elimination procedure. For backward elimination the 1-step error rate for incorrectly specifying heterogeneous *π *was 6%, and for incorrectly specifying homogenous *π *was 14%, indicating a greater tendency towards too simple a model. For AIC and AICc, the misspecification of *π *was almost entirely for too simple a model. Note that most of these errors were made in the 90:10 datasets, suggesting that misspecification of codon frequency heterogeneity is mainly due to large empirical estimation-errors of codon frequencies due to the insufficient information of small partitions. Thus power is lowest to identify heterogeneity in codon frequencies when a partition consists of a small number of codon sites. We anticipate that power also will be low in larger partitions of real datasets where the difference among partitions is not as great as in our simulations.

Next we investigated the possibility of tuning the cut-off *p*-value of the backward elimination procedure to improve the accuracy of model specification. We evaluated accuracy for cut-off *p*-values of 0.01, 0.001 and 0.0001. Substantial improvements were obtained, with average accuracy increasing from 78% (under the original cut-off *p*-value of 0.05) to 83%, 87% and 88% (Table 3) respectively. Under a cut-off value of 0.0001, 39 models were misspecified, with 33 being too simple with respect to codon frequencies in the 90:10 datasets. Among the 6 remaining misspecified models, 4 were too complex for *ω *and 2 were too complex for *π*. All but one of the misspecified models under cut-off *p *= 0.0001 were one-step errors. Based on these findings we used a cut-off *p *= 0.0001 in our application of these models to real data.

### Abalone sperm lysin gene

Abalone sperm lysin is a reproductive protein well known for rapid evolution under strong diversifying selection [22]. We partitioned the lysin dataset into the same set of 46 buried sites and 88 solvent-exposed sites as in Yang and Swanson [4] and applied backward elimination (cut-off *p *= 0.0001), AIC and AICc to select among the full set of fixed-effect models. Under backward elimination, we used the likelihood ratio test to compare FE1, which assumes different *κ*, *ω*, *c*, and *π*'s for buried and exposed sites, with those nested models at the next level in Figure 1 (FE2, FE3, FE5 and FE9). Each model at the next level assumes one of these four parameters (*κ*, *ω*, *c *or *π*) is homogeneous among site classes. FE9 assumes homogenous *κ *for both buried and solvent-exposed sites, and the likelihood ratio statistic comparing FE1 against FE9 is 2 × (4474.75-4473.88) = 1.74, which is not significant (d.f. = 1; *p *= 0.1871). As all other LRTs at this level are significant, we simplified our model according to *κ *and compared FE9 to those models nested at the next level in Figure 1 (FE10, FE11 and FE13). As subsequent LRTs involving FE9 were significant, model FE9 was selected by backward elimination. We note that even when we use a cut-off *p *= 0.05, we still select FE9 by backward elimination. Table 4 illustrates that FE9 is also selected by using AIC or AICc.

Yang and Swanson [4] conducted LRTs of the subset of models shown in Table 1 and found that Model E (FE1) provided the best fit to the lysin data. FE1 and FE9 are qualitatively similar in suggesting heterogeneity in *ω*, *c *and *π*'s among the buried (b) and solvent exposed (e) sites. Moreover, these models provide similar quantitative estimates of the strength of selection and rate of evolution in these two partitions (FE1: *ω*_b _= 0.45, *ω*_e _= 1.28, *c*_b _= 1, *c*_e _= 2.54; FE9: *ω*_b _= 0.43, *ω*_e _= 1.31, *c*_b _= 1, *c*_e _= 2.56). Both models suggest that buried sites are evolving under strong purifying selection and exposed sites under diversifying selection. Note that Yang and Swanson [4] used a model that specified heterogeneous *κ*'s (FE1), as it was not possible to test for heterogeneity in *κ*'s independently of *ω*'s (Table 1). As estimates of *ω *were very similar under FE1 and FE9, and *κ*'s for partitions under FE1 were very similar (*κ*_b _= 1.9 and *κ*_e _= 1.5), the use of FE1 was not problematic in this case.

### Components of the *Listeria *flagellar system

*Listeria *are gram positive rod shaped bacteria which are motile between 4°C and 30°C, and grow in a wide range of pHs, temperatures, and osmotic pressures. The natural habitat of *Listeria *is thought to be soil rich in decaying matter; however, *Listeria monocytogenes *is an important food-borne pathogen of humans and animals capable of both a free-living and intracellular lifestyle. Interestingly, the motility of *Listeria monocytogenes *is thermoregulated, being reduced above 30°C, and completely shut down above 37°C [23], temperatures which correspond to their host intracellular environment. The consensus opinion is that the shut down of expression of flagellar related proteins, thereby shutting down motility, is an adaptation to avoid recognition by the hosts innate immune system [24]. Specifically, recognition of the flagellin protein, a product of the *flaA *gene, activates the host inflammatory responses through Toll-like receptor 5 (TLR5) [25].

Twenty-eight genes encoding putative flagellar related proteins, including *flaA*, are located together in the genome of *Listeria*. Several proteins having functions unrelated to flagellar machinery, or unknown functions, also are encoded in this region of the genome. We analysed the genes from this region with three issues in mind: (i) to test for heterogeneous evolutionary dynamics among genes, (ii) to examine the evolutionary dynamics of proteins with unknown function and determine if they have any similarities to flagellar machinery proteins or proteins having unrelated functions, and (iii) to compare selection pressures on *flaA *with other genes known to encode flagellar related proteins. We note that thermoregulation of motility is not always perfect, thereby raising the possibility that the host's innate immune system is occasionally able to recognize flagellin [24]. This would set up selection pressure for a "co-evolutionary chase" between host and pathogen, leading to an elevated rate of nonsynonymous evolution in *flaA*.

Our dataset was comprised of 37 of the 43 genes (lmo0675 – lmo0717) located contiguously within the genomes of 5 lineages of *Listeria*. Two genes (lmo0684 and lmo0711) were excluded because their gene trees were incompatible with the genome-tree. Four genes (lmo0677, lmo0698, lmo0709 and lmo0712) were excluded because they were less than 100 codons long. Next we partitioned the genes according to functional category: (i) flagellar machinery (7973 codons), (ii) non-flagellar functions (1427 codons) and (iii) unknown functions (2308 codons). We then applied backward elimination (cut-off *p *= 0.0001), AIC and AICc to select among the full set of fixed-effect models (Table 5). Unlike the lysin example above, the model selected by backward elimination (FE9) differed from the model selected by AIC and AICc (FE10). Both FE9 and FE10 indicate heterogeneity in *c *and *ω*, and homogeneity in *κ *among partitions. They differ in that FE9 specifies heterogeneous codon frequencies and FE10 does not. Clearly, the genes in this region of the *Listeria *genome are subject to heterogeneous evolutionary dynamics.

Interestingly, genes encoding proteins of unknown function had levels of selection pressure (FE9: *ω *= 0.036; FE10: *ω *= 0.038) highly similar to genes encoding proteins known to comprise the flagellar machinery (FE9: *ω *= 0.016; FE10: *ω *= 0.018), whereas those genes that do not encode components of the flagellar machinery were evolving under substantially higher relative rates of amino acid substitution (FE9: *ω *= 0.11; FE10: *ω *= 0.11). Genes encoding several components of the flagellar machinery (FliO, FliJ, FliT, FlgM, and FliK) are present in other bacilli but unaccounted for in *Listeria*. We used BLAST to compare the present set of unknown proteins to other bacilli and found one case (lmo0715) that was similar to a known flagellar protein (FliH). We note that the KEGG database [26] has annotated lmo0715 as a putative flagellar assembly protein. Based on genome location and levels of selection pressure, we suggest that the "unknown" genes in this dataset represent the best candidates for the unaccounted components of the *Listeria *flagellar machinery. Genes that evolve at high rates can be difficult to identify [27]; however this is not the case here, as estimates of *ω *indicate a relatively slow rate of nonsynonymous evolution. If these genes indeed encode the missing components of the *Listeria *flagellar machinery, we speculate an ancestor of *Listeria *might have acquired them via an LGT event.

The rapidly-evolving non-flagellar genes encoded (i) a protein involved in regulating chemotaxis (lmo0691), (ii) a chemotaxis-related sensory protein (lmo0692), (iii) a cell surface protein (lmo0701), and (iv) a phage-related protein similar to transglycosylase (lmo0717). To further investigate the evolutionary dynamics of these genes we applied the full set of fixed-effect models to them, with each having a unique partition. Again, the model selected by backward elimination (FE9) differed from the model selected by AIC and AICc (FE10). Results under FE9 (Table 6) and FE10 are consistent in suggesting heterogeneous *ω *among them, with one gene, the cell surface protein, having a substantially higher relative rate of nonsynonymous evolution. Interestingly, a genome wide survey of *Listeria *genes reveals that, in general, cell-surface proteins exhibit accelerated evolutionary rates as compared to housekeeping genes (unpublished data).

Lastly, we investigated the evolutionary dynamics of *flaA *as compared to those genes known to encode flagellar related proteins. We applied the full set of models to this subset of proteins, with *flaA *having a unique partition and the remaining 23 proteins placed in a second partition. In this case backward elimination, AIC and AICc selected model FE13. This indicates that, despite heterogeneity in both *c *and *π*, selection pressure on *flaA *does not differ significantly from the average for genes encoding a flagellar related protein. This result supports the hypothesis that thermoregulation of motility remains an effective adaptation to avoid recognition by the host's innate immune system [24], despite sometimes less than perfect control over gene expression.

## Discussion

The simulation results show that under a cut-off *p*-value = 0.05 the likelihood ratio test is more accurate than AIC and AICc. With the exception of the *π *parameters, AIC and AICc chose overly complex models more frequently than did the backward elimination procedure. For the *π *parameters, AIC and AICc chose overly simple models more frequently than did backward elimination. The difference lies in the different cut-off values that are used to penalize the more complex model. Take the heterogeneity test of *κ *as an example (df = 1), the LRT statistic is defined as twice the difference in log likelihood values between a pair of nested models: Λ = 2 × (ln *L*( | *x*) - ln *L*(*θ*_0 _| *x*)). Based on the LRT under a significance level of 0.05, we reduce the complexity of the model if Λ is smaller than the critical value 3.84. Under AIC we choose a simpler model only when Λ < 2; hence, AIC tends toward more complex models. However when we reduce a model by more than 7 parameters (e.g. homogeneous *π*'s versus heterogeneous *π*'s, with d.f. = 9), the critical value becomes 16.92 for the LRT, which is less than the critical value of Λ under AIC, 18. In this case, AIC will select the same or simpler model than the LRT. Note that AICc compares Λ with 2 × *k *× *n/(n-2) *which is always greater than 2 × *k *used by AIC, hence AICc will always choose the same or simpler model than AIC. This property of AICc had only a small effect on the results of model selection, as AICc performed only slightly better than AIC but substantially worse than backward elimination.

LRT statistics involving parameters such as *ω *appear to be asymptotically *χ*^2 ^distributed for random-effect codon models; such models employ a parametric distribution (*e.g.*, the *β *distribution of Models M7 and M8 [2]) to accommodate among site variability in the *ω *ratio. However, Aagaard and Phillips [8] reported that for comparisons of Yang and Swanson's [4] models C and E (FE13 and FE1 in Figure 1), the empirical distribution of LRT statistics deviated from the expected *χ*^2 ^distribution, leading to a type I error rate in excess of that specified by the level of the test. The results of our simulation study suggest this bias might affect all tests in Figure 1 involving parameters *κ*, *ω *and *c*. Several authors have noted that LRT statistics derived from models that employ empirical estimates of nucleotide or codon frequencies might not be well approximated by a *χ*^2 ^distribution [8,28]. Moreover, when Aagaard and Phillips [8] repeated their simulation study under equal codon frequencies and computed LRT statistics by using models with frequency parameters fixed to the true values (*π*_*i *_= 1/61), they found that the LRT statistics matched the *χ*^2 ^expectation. Aagaard and Phillips [8] suggested that the approximation of codon frequencies is the source of the observed bias in the LRT.

Indeed, empirical estimates do not satisfy the requirements of LRT [21], and consequently the backward elimination procedure. To further investigate the impact of empirical estimates on model selection, we reanalysed all the simulated datasets under the true codon frequencies; *i.e*., those used to generate the data. We note that for a given dataset the empirical codon frequencies yielded higher likelihood scores than did the true frequencies. This was expected, as empirical estimation will "pick up" some of the sampling errors in each simulation replicate. We found that the accuracy and bias of backward elimination, AIC and AICc under the true codon frequencies were identical to those when empirical codon frequencies were used. This suggests that bias in the model selection procedures used here did not arise from empirical estimation of *π*'s alone.

There are several possible explanations for the bias of all three model selection methods in the direction of greater complexity for one of *ω*, *κ *or *c*. One possibility is that potential non-independence among the values for different parameters means that AIC might not be a good approximation to the Kullback-Leibler divergence, and that the requirements for the *χ*^2 ^approximation might not be met for the LRT, thus the degree of freedom is not accurate. Also, backward elimination may find a local optimum solution. Under backward elimination the cut-off *p*-value is subjectively decided before the tests, leading to the possibility that the procedure will stop too early and suggest an overly complex model. This phenomenon is sometimes seen in the regression context. Clearly, these issues require further attention; in the mean time we explored the possibility of tuning the cut-off *p*-value in order to improve the performance of backward elimination (see also [8]). After evaluating several cut-off values for *p*, we found that a substantial improvement in performance was obtained by using *p *= 0.0001. Moreover, we found that by using this cut-off, nearly all the tendency to select an overly complex model for *ω*, *κ *or *c *was avoided, and that errors for selecting overly-simplistic models happen mostly for datasets where one of the partitions was comprised of a very small number of codon sites.

Our application of fixed-effect models to real data was encouraging, having uncovered previously unrecognized heterogeneity among *Listeria *genes and among sites within the abalone sperm lysin gene. However, if the objective is only to identify individual positive selection sites within a gene, the *a priori *structural information is not likely to serve as a perfect proxy for those partitions most relevant to differences in selection pressures. For example, Yang and Swanson [4] showed that the exposed sites of lysin likely include both conserved and positively selected codon positions. Hence, averaging *ω *over all sites in the exposed partition yields a reduced estimate of positive selection pressure. We note this effect was the same under the best-fit model, FE9, as under FE1 (Model E) used by Yang and Swanson [4]. We agree with Yang and Swanson [4] in anticipating that the power of random-effect models to test the strength and direction of selection pressure at sites within genes will be greater than fixed-effect models in most cases.

If the objective is to investigate heterogeneous evolution among genes, as in genome-scale analyses, then fixed-effect models are useful. The present set of models represents only a small step towards genome-scale evolutionary models. For example, decoupling synonymous and nonsynonymous rates, as in the random-effect model of Kosakovsky Pond and Muse [29], would allow users freedom to model gradients in synonymous substitution rates along a genome while allowing independent variability in nonsynonymous rates among genes. Yang and Swanson [4] made several suggestions, including the intriguing idea of enforcing a molecular clock for synonymous changes and leaving nonsynonymous changes unconstrained. We predict that joining fixed-effect codon models and data-mining methods to obtain new methods analogous to model based clustering [30,31] could provide extremely useful tools for genome-scale data analysis. Lastly, there is growing interest in both the performance of codon models [32,33] and the impact of heterogeneity among genes [34,35] in multi-gene phylogenetic analysis; improved ability to model among-gene heterogeneity at the codon level could improve their utility for comparing alternative phylogenomic hypotheses.

## Conclusion

Random- and fixed-effect codon models have unique advantages and disadvantages. Random-effect models are desirable when there is no *a priori *knowledge by which sites might be partitioned, or when only a few sites are expected to comprise a partition of interest (but see [5]). Their disadvantage is that models for heterogeneity among sites in features such as the transition to transversion ratio (*κ*) and equilibrium codon frequencies (*π*) are unavailable. Fixed-effect models are desirable when data partitions are known to exhibit significant heterogeneity in parameters such as *c*, *κ *or *π*, or when a statistical test of such heterogeneity is desired. The disadvantage here is that any uncertainty in the site partition is not accommodated.

The growing importance of phylogenomics and metagenomics (*e.g.*, [36,37]) will lead to a greater need for models suitable for testing hypotheses, and estimating rates and patterns of evolution, in large multi-gene datasets. Although considerable development remains to be done, we believe the present set of models will find many useful applications provided that results are interpreted with the inherent limitations of the methods in mind. In particular we note: (i) power can be low (see also [8]), particularly when partitions are small, (ii) the accuracy of the partitions may influence the results of model specification and (iii) the tree topology is assumed to be known without error. For the time being we make the following recommendations: (i) select among models by using backward elimination rather than AIC or AICc, (ii) use a stringent cut-off *p*-value; *p *= 0.0001 seems appropriate, and (iii) sensitivity analysis should be included in an investigation. Sensitivity of results should be investigated for robustness to tree topology and model of codon frequencies. We note that by using Akaike weights [16] to quantify the evidence in favour of a model, estimates of parameters could be obtained that accommodate model uncertainties (*e.g*., [19]). Where practical, we recommend that sensitivity to alternative data partitions also should be explored. Lastly, any complex model can have convergence problems or implementation errors; one must always inspect the parameter estimates for atypical results. With thoughtful application, fixed-effect codon models should provide a useful tool for large scale multi-gene analyses.

## Methods

### Fixed-effect models of codon evolution

The basic codon model [9,10] assumes that the process of substitutions from one codon to another is a Markov process, where the next codon state only depends on the present state, and not on any past state. The element *P*_*ij*_*(t) *in the transition matrix *P(t) *gives the probability of going from codon *i *to codon *j *during time *t*. Because they do not occur within a functional protein-coding gene, the three stop codons, UAA, UAG and UGA, are excluded. The transition matrix *P(t) *can be calculated by *P(t) *= *e*^*Qt*^, where *Q *= *{q_*ij*_} *is a 61 × 61 rate matrix. The element *q*_*ij *_denotes the instantaneous substitution rate from codon *i *to codon *j *as follows:



When a change among codons involves a transition, the rate is multiplied by *κ*, the transition/transversion rate ratio. In the same way, if the substitution is nonsynonymous, the rate is multiplied by *ω*, the nonsynonymous/synonymous rate ratio. Usage of codons within a gene can also be highly biased, and consequently the rate is multiplied by the equilibrium frequency of the targeted codon *π*_*j*_, which is assumed to remain unchanged between generations of the evolutionary process. Given prior information by which sites can be partitioned into classes, parameters such as *ω*, *κ *and *π *can be allowed to differ among partitions, with different *Q *matrices used for different partitions [4]. Independence among all codon sites is assumed; hence the log likelihood of the complete dataset is the sum of the log likelihood of each site [4].

For all fixed-effect codon models described in this paper (Table 2) the tree topology is fixed *a priori *and, with the exception of the codon frequencies, the parameter values are estimated by numerical maximization of the likelihood function. Codon frequencies are empirically estimated from the data. For parameters heterogeneous among partitions, we use the maximum likelihood estimation implemented by Yang and Swanson [4]. For the models with identical substitution rate, we simply fix the branch length ratio *c *at 1 across partitions. For models with homogeneous *κ *or *ω*, we use an algorithm similar to the Expectation-Maximization (EM) algorithm [38]. Let's take a single parameter, say *ω*, as an example of a homogenous parameter. We first independently estimate the parameters in each partition by maximum likelihood. At the "E-step" of the algorithm we obtain the weighted average of *ω *over all partitions, where the weight is given by the proportion of codon sites in the corresponding partition. At the "M-step" we re-estimate parameters heterogeneous over partitions after fixing *ω *to its weighted average value. Following this we run the "E-step" by fixing the parameters assumed to be heterogeneous, and re-estimating *ω *from each partition; an updated estimate of *ω *is again obtained as the weighted average. The E- and M-steps are run iteratively until successive estimates of *ω *converge.

### Model selection among a set of related fixed-effect models

Backward elimination reduces the most complex codon model, shown at the top of Figure 1, to a simpler one in a stepwise fashion. We begin from model FE1, which assumes different *κ*, *ω*, *c*, and *π*'s for different site classes, and then compare it with simpler models which assume one of these four parameters to be homogeneous (see next level in Figure 1). For example, if the hypothesis of homogeneous *κ *is not rejected, we will go to FE9 at the next level in Figure 1, which assumes different *ω*, *c*, *π*'s but the same *κ *for different site classes. We then compare FE9 with its nested models at the next level (see FE10, FE11 and FE13 in Figure 1). If more than one homogenous model cannot be rejected by the LRTs at a given level, the backward elimination procedure will choose the model with the largest *p*-value in the LRT.

Akaike Information Criterion (AIC) is based on minimizing the expected Kullback-Leibler divergence [39,40]; *AIC *= -2 × ln *L*( | *x*) + 2 × *k*, where *k *denotes the number of free parameters in the candidate model. For small samples, the AIC is corrected by a second order bias adjustment in the regression and time series settings in order to avoid over-fitting, let *n *denote the number of observations [17]: . This adjustment places a heavier penalty on the number of parameters when the number of observations is not much larger than the number of parameters; thus AICc tends to choose a simpler model than AIC. We note that the basis for this correction is not expected to hold outside of the regression and time-series settings; however, because we desired a correction for small samples we evaluated the performance of AICc by numerical simulations. In our analysis *k *denoted the number of parameters in a given model (Table 2) and *n *denoted the number of codon sites. For both AIC and AICc, the model with the smallest score is chosen as the ideal model.

The full set of 16 fixed-effect models (Figure 1) is implemented in a modified version of codeml that we make freely available [41]. The original version of codeml (3.14b) is one of several programs in the PAML package [42] distributed by Ziheng Yang [43].

### Simulated and real sequence data

We simulated codon evolution by using the "evolver" program of the PAML package [42]. There were 16 different scenarios, based on the heterogeneity or homogeneity of four parameters (Table 2) among two data partitions. Parameter values are shown in Table 7. We also varied the ratio of the number of codon sites contained in each partition (50:50, 70:30 or 90:10). Data were simulated independently in two partitions and then concatenated to obtain a composite dataset. Each composite dataset contained 2000 codons for 16 species. A total of 432 composite datasets were possible; *i.e*., the product of 2 options on *κ*, 3 options on *c*, 4 options on *π*, 6 options on *ω *and 3 partition proportions. However, as we had two options for heterogeneous rates (1:3 or 1:9) and one possibility for the homogeneous rates (1:1) we made an adjustment to obtain similar number of datasets for each of the 16 scenarios. Specifically, we simulated under only 4 of the options on *ω *(first and second rows for selection pressure in Table 7) when rates were assumed to be heterogeneous among partitions (*c*_2 _= 3 and *c*_2 _= 9). Thus 2(*κ*) × 2(*c*) × 4(*π*) × 2(*ω*) × 3(sites proportion) = 96 possibilities were not included in our simulation. This strategy provided a grand total of 336 composite datasets for evaluating the performance of the different model selection strategies.

The first real dataset was comprised of sequences for the sperm lysin gene from 25 species of abalone. This is one of the original test cases of Yang and Swanson's paper [4], and it is distributed online by Ziheng Yang as part of the PAML package [43]. Note that this lysin dataset and phylogeny is the same as those from [44], except that a single site containing an alignment gap was excluded. The second real dataset was comprised of 37 genes located within the genomic region of *Listeria *bacteria that encode the components of the flagellar system (lmo0675 – lmo0717). We note that this region includes several proteins of unknown function, and for comparative purposes we included them in our dataset. Genes were partitioned according to functional categories of the ListiList database [45]. The sequence alignments, phylogenetic trees and gene ontologies for this multi-gene dataset is available online [46]. Although multi-gene datasets can be much larger than ours, it represents both a real biological problem and serves as an illustration of the types of data upon which these techniques can be used.

## Authors' contributions

All authors contributed to the interpretation of results, were involved in the drafting and revising of the manuscript, and have read and approved the final version. LB developed the computer code. KD contributed the design and assembly of the *Listeria *data analysis, and made substantial contributions to interpretation of those data.

## Figures and Tables

**Table 1:** Fixed-effect models implemented by Yang and Swanson [4].

Model code	Parameters for partitions	Number of Parameters
A	same branch lengths, *κ*, *ω*, and *π*'s	*b*+2+9
B	different but proportional branch lengths, same *κ*, *ω*, and *π*'s	*b*+(*g*-1)+2+9
C	different but proportional branch lengths, same *κ*, *ω*, and different *π*'s	*b*+(*g*-1)+2+g×9
D	different but proportional branch lengths, different *κ*, *ω*, and same *π*'s	*b*+(*g*-1)+*g*×2+9
E	different but proportional branch lengths, different *κ*, *ω*, and *π*'s	*b*+(*g*-1)+*g*×2+*g*×9
F	different branch lengths, *κ*, *ω*, and *π*'s	*g*×(*b*+2+9)

The number of parameters is computed under the F3 × 4 method of estimating codon frequencies. *b *denotes the number of branches in the tree. *g *denotes the number of site classes. When models employ empirical estimates of each codon frequency (F61 method) the number of model parameters increases by 51 for models with homogenous *π*'s, and by 51 × *g *for models with heterogeneous *π*'s among partitions.

**Table 2:** An expanded set of fixed-effect models.

	Parameters heterogeneous among partitions				
		
New code	*κ*	*ω*	*c*	*π*	Number of parameters
1 (E)	Yes	Yes	Yes	Yes	*b*+12*g*-1
2 (D)	Yes	Yes	Yes	No	*b*+3*g*+8
3	Yes	Yes	No	Yes	*b*+11*g*
4	Yes	Yes	No	No	*b*+2*g*+9
5	Yes	No	Yes	Yes	*b*+11*g*
6	Yes	No	Yes	No	*b*+2*g*+9
7	Yes	No	No	Yes	*b*+10*g*+1
8	Yes	No	No	No	*b*+*g*+10
9	No	Yes	Yes	Yes	*b*+11*g*
10	No	Yes	Yes	No	*b*+2*g*+9
11	No	Yes	No	Yes	*b*+10*g*+1
12	No	Yes	No	No	*b*+*g*+10
13 (C)	No	No	Yes	Yes	*b*+10*g*+1
14 (B)	No	No	Yes	No	*b*+*g*+10
15	No	No	No	Yes	*b*+9*g*+2
16 (A)	No	No	No	No	*b*+11

Number of parameters is for the F3 × 4 method of estimating codon frequencies. *b *and *g *denote the number of branches and the number of site classes, respectively. Letters in parentheses indicate the model codes formerly used by Yang and Swanson [4].

**Table 3:** Accuracy of model selection under backward elimination, AIC and AICc. Letters in parentheses indicate the model codes formerly used by Yang and Swanson [4].

		Backward elimination			
				
Model	Heterogeneous parameters	*p *= 0.05	*p *= 0.0001	AIC	AICc
1 (E)	*κ*, *ω*, *c*, *π's*	100%	96%	91.7%	91.7%
2 (D)	*κ*, *ω*, *c*	92%	100%	91.7%	100.0%
3	*κ*, *ω*, *π's*	61%	67%	38.9%	33.3%
4	*κ*, *ω*	94%	100%	77.8%	83.3%
5	*κ*, *c*, *π's*	88%	92%	62.5%	66.7%
6	*κ*, *c*	79%	100%	75.0%	75.0%
7	*κ*, *π's*	67%	67%	44.4%	44.4%
8	*κ*	83%	100%	55.6%	55.6%
9	*ω*, *c*, *π's*	71%	83%	58.3%	58.3%
10	*ω*, *c*	79%	96%	75.0%	75.0%
11	*ω*, *π's*	50%	67%	38.9%	38.9%
12	*ω*	89%	100%	66.7%	66.7%
13 (C)	*c*, *π's*	83%	88%	62.5%	58.3%
14 (B)	*c*	63%	88%	58.3%	58.3%
15	*π's*	56%	61%	38.9%	38.9%
16 (A)	none	83%	100%	38.9%	55.6%
overall		78%	88%	63%	64%

**Table 4:** Likelihood scores, parameter estimates, ΔAIC and ΔAIC_C _scores for the abalone sperm lysin gene under codon models with two fixed partitions.

		Parameter estimates			
			
Model	ℓ	*c*	*ω*	*κ*	ΔAIC (ΔAIC_C_)
1 (E)	-4473.88	(*c*_1 _= 1) *c*_2 _= 2.54	*ω*_1 _= 0.45 *ω*_2 _= 1.28	*κ*_1 _= 1.93 *κ*_2 _= 1.51	0.3 (6.9)
2 (D)	-4532.12	(*c*_1 _= 1) *c*_2 _= 2.69	*ω*_1 _= 0.39 *ω*_2 _= 1.25	*κ*_1 _= 1.73 *κ*_2 _= 1.51	98.7 (54.2)
3	-4535.64	(*c *= 1)	*ω*_1 _= 0.37 *ω*_2 _= 1.27	*κ*_1 _= 2.27 *κ*_2 _= 1.49	121.8 (121.8)
4	-4603.44	(*c *= 1)	*ω*_1 _= 0.33 *ω*_2 _= 1.26	*κ*_1 _= 2.03 *κ*_2 _= 1.5	239.4 (190.2)
5	-4486.60	(*c*_1 _= 1) *c*_2 _= 2.56	*ω *= 1.11	*κ*_1 _= 2.53 *κ*_2 _= 1.47	23.7 (23.7)
6	-4548.86	(*c*_1 _= 1) *c*_2 _= 2.72	*ω *= 1.07	*κ*_1 _= 2.14 *κ*_2 _= 1.47	130.2 (81.0)
7	-4550.36	(*c *= 1)	*ω *= 1.12	*κ*_1 _= 3.19 *κ*_2 _= 1.45	149.2 (142.9)
8	-4622.36	(*c *= 1)	*ω *= 0.93	*κ*_1 _= 2.55 *κ*_2 _= 1.42	275.2 (221.6)
9	-4474.75	(*c*_1 _= 1) *c*_2 _= 2.56	*ω*_1 _= 0.43 *ω*_2 _= 1.31	*κ *= 1.64	0 (0)
10	-4532.38	(*c*_1 _= 1) *c*_2 _= 2.70	*ω*_1 _= 0.38 *ω*_2 _= 1.26	*κ *= 1.58	97.3 (48.1)
11	-4538.32	(*c *= 1)	*ω*_1 _= 0.34 *ω*_2 _= 1.32	*κ *= 1.73	125.1 (118.8)
12	-4604.91	(*c *= 1)	*ω*_1 _= 0.32 *ω*_2 _= 1.29	*κ *= 1.68	240.3 (186.6)
13 (C)	-4490.07	(*c*_1 _= 1) *c*_2 _= 2.61	*ω *= 1.00	*κ *= 1.60	28.6 (22.3)
14 (B)	-4549.99	(*c*_1 _= 1) *c*_2 _= 2.76	*ω *= 0.95	*κ *= 1.55	130.5 (76.8)
15	-4561.36	(*c *= 1)	*ω *= 0.96	*κ *= 1.95	169.2 (156.7)
16 (A)	-4627.03	(*c *= 1)	*ω *= 0.96	*κ *= 1.58	282.6 (224.6)

Parameters in parentheses are fixed. Partition 1 contained the buried sites and partition 2 contained the solvent exposed sites. ΔAIC_*i *_= AIC_*i *_- min AIC, and ΔAIC_C*i *_= AIC_C*i *_- min AIC_C_.

**Table 5:** Likelihood scores, parameter estimates, ΔAIC and ΔAIC_C _scores for genes located in the regions of the *Listeria *genome encoding putative flagellar related proteins.

		Parameter estimates			
			
Model	ℓ	*c*	*ω*	*κ*	ΔAIC (ΔAIC_C_)
1 (E)	-64965.25	(*c*_1 _= 1) *c*_2 _= 1.18 *c*_3 _= 1.42	*ω*_1 _= 0.02 *ω*_2 _= 0.03 *ω*_3 _= 0.10	*κ*_1 _= 1.97 *κ*_2 _= 1.59 *κ*_3 _= 1.62	22.2 (28.2)
2 (D)	-65076.39	(*c*_1 _= 1) *c*_2 _= 1.21 *c*_3 _= 1.37	*ω*_1 _= 0.02 *ω*_2 _= 0.04 *ω*_3 _= 0.10	*κ*_1 _= 1.91 *κ*_2 _= 1.76 *κ*_3 _= 1.62	0.5 (0.6)
3	-65000.40	(*c *= 1)	*ω*_1 _= 0.02 *ω*_2 _= 0.03 *ω*_3 _= 0.11	*κ*_1 _= 2.02 *κ*_2 _= 1.56 *κ*_3 _= 1.56	88.5 (94.3)
4	-65107.87	(*c *= 1)	*ω*_1 _= 0.02 *ω*_2 _= 0.04 *ω*_3 _= 0.11	*κ*_1 _= 1.97 *κ*_2 _= 1.74 *κ*_3 _= 1.57	59.5 (59.5)
5	-65106.46	(*c*_1 _= 1) *c*_2 _= 1.21 *c*_3 _= 1.59	*ω *= 0.03	*κ*_1 _= 2.10 *κ*_2 _= 1.54 *κ*_3 _= 1.22	300.7 (306.5)
6	-65219.99	(*c*_1 _= 1) *c*_2 _= 1.25 *c*_3 _= 1.51	*ω *= 0.03	*κ*_1 _= 2.06 *κ*_2 _= 1.68 *κ*_3 _= 1.21	283.7 (283.7)
7	-65162.07	(*c *= 1)	*ω *= 0.03	*κ*_1 _= 2.18 *κ*_2 _= 1.50 *κ*_3 _= 1.14	407.9 (413.5)
8	-65269.12	(*c *= 1)	*ω *= 0.03	*κ*_1 _= 2.14 *κ*_2 _= 1.65 *κ*_3 _= 1.15	378.0 (377.9)
9	-64969.44	(*c*_1 _= 1) *c*_2 _= 1.20 *c*_3 _= 1.44	*ω*_1 _= 0.02 *ω*_2 _= 0.04 *ω*_3 _= 0.11	*κ *= 1.86	26.6 (32.4
10	-65078.13	(*c*_1 _= 1) *c*_2 _= 1.22 *c*_3 _= 1.38	*ω*_1 _= 0.02 *ω*_2 _= 0.04 *ω*_3 _= 0.11	*κ *= 1.85	0 (0)
11	-65007.34	(*c *= 1)	*ω*_1 _= 0.02 *ω*_2 _= 0.04 *ω*_3 _= 0.12	*κ *= 1.89	98.4 (104.1)
12	-65111.31	(*c *= 1)	*ω*_1 _= 0.02 *ω*_2 _= 0.04 *ω*_3 _= 0.11	*κ *= 1.89	62.4 (62.4)
13 (C)	-65124.90	(*c*_1 _= 1) *c*_2 _= 1.24 *c*_3 _= 1.66	*ω *= 0.03	*κ *= 1.83	333.6 (339.2)
14 (B)	-65235.39	(*c*_1 _= 1) *c*_2 _= 1.27 *c*_3 _= 1.57	*ω *= 0.03	*κ *= 1.83	310.5 (310.5)
15	-65190.76	(*c *= 1)	*ω *= 0.03	*κ *= 1.81	461.5 (466.8)
16 (A)	-65292.90	(*c *= 1)	*ω *= 0.03	*κ *= 1.83	421.5 (421.4)

Parameters in parentheses are fixed. Partition 1 contained genes known to encode components of the flagellar machinery (7973 codons). Partition 2 contained genes encoding proteins with unknown functions (2308 codons). Partition 3 contained genes encoding proteins with non-flagellar functions (1427 codons). ΔAIC_*i *_= AIC_*i *_- min AIC, and ΔAIC_C*i *_= AIC_C*i *_- min AIC_C_.

**Table 6:** Likelihood scores and parameter estimates obtained under the model selected by backward elimination for subsets of the genes located in the regions of the *Listeria *genome encoding putative flagellar related proteins.

			Parameter estimates		
			
Dataset & numbered partitions	Model	ℓ	*c*	*ω*	*κ*
Rapidly-evolving non-flagellar genes [4 genes]	FE9	-8528.24			
1. Chemotaxis regulatory protein			(*c*_1 _= 1)	*ω*_1 _= 0.0001	*κ*_1 _= 1.77
2. Chemotaxis-related sensory protein			*c*_2 _= 1.22	*ω*_2 _= 0.03	*κ*_2 _= *κ*_1_
3. Cell surface protein			*c*_3 _= 5.35	*ω*_3 _= 0.17	*κ*_3 _= *κ*_1_
4. Phage-related, similar to transglycosylase			*c*_4 _= 3.77	*ω*_4 _= 0.05	*κ*_4 _= *κ*_1_
					
Flagellar related genes [24 genes]	FE13	-42796.43			
1. *flaA *gene			(*c*_1 _= 1)	*ω*_1 _= 0.017	*κ*_1 _= 1.96
2. 23 other genes			*c*_2 _= 0.14	*ω*_2 _= *ω*_1_	*κ*_2 _= *κ*_1_

Number within square brackets is the number of genes in the dataset. Parentheses indicate a model parameter with a fixed value.

**Table 7:** Parameter values used in simulations of two-partition datasets.

	Homogeneous parameter values		Heterogeneous parameter values	
		
	partition 1	partition 2	partition 1	partition 2
Rates	*c*_1 _= 1	*c*_2 _= 1	*c*_1 _= 1 *c*_1 _= 1	*c*_2 _= 3 *c*_2 _= 9
Selection pressure	*ω*_1 _= 0.25 *ω*_1 _= 0.75 *ω*_1 _= 2.25	*ω*_2 _= 0.25 *ω*_2 _= 0.75 *ω*_2 _= 2.25	*ω*_1 _= 0.25 *ω*_1 _= 0.75 *ω*_1 _= 0.25	*ω*_2 _= 0.75 *ω*_2 _= 2.25 *ω*_2 _= 2.25
Ts/Tv ratio	*κ*_1 _= 1.25	*κ*_2 _= 1.25	*κ*_1 _= 1.25	*κ*_2 _= 3.75
Codon frequencies	*π*_*i*__1 _= 1/61 *π*_*i*__1 _= lysin	*π*_*i*__2 _= 1/61 *π*_*i*__2 _= lysin	*π*_*i*__1 _= 1/61 *π*_*i*__1 _= lysin	*π*_*i*__2 _= lysin *π*_*i*__2 _= 1/61

The lysin gene was used to obtain empirical estimates of codon frequencies, this is indicated in the table by *π*_*i *_= lysin.

## References

[B1] Nielsen R, Yang Z (1998). Likelihood models for detecting positively selected amino acid sites and applications to the HIV-1 envelope gene. Genetics.

[B2] Yang Z, Nielsen R, Goldman N, Pedersen AM (2000). Codon-substitution models for heterogeneous selection pressure at amino acid sites. Genetics.

[B3] Suzuki Y, Gojobori T (1999). A method for detecting positive selection at single amino acid sites. Mol Biol Evol.

[B4] Yang Z, Swanson WJ (2002). Codon-substitution models to detect adaptive evolution that account for heterogeneous selective pressures among site classes. Mol Biol Evol.

[B5] Kosakovsky Pond SL, Frost SD (2005). Not so different after all: a comparison of methods for detecting amino acid sites under selection. Mol Biol Evol.

[B6] Massingham T, Goldman N (2005). Detecting amino acid sites under positive selection and purifying selection. Genetics.

[B7] Clark AG, Glanowski S, Nielsen R, Thomas PD, Kejariwal A, Todd MA, Tanenbaum DM, Civello D, Lu F, Murphy B, Ferriera S, Wang G, Zheng X, White TJ, Sninsky JJ, Adams MD, Cargill M (2003). Inferring nonneutral evolution from human-chimp-mouse orthologous gene trios. Science.

[B8] Aagaard JE, Phillips P (2005). Accuracy and power of the likelihood ratio test for comparing evolutionary rates among genes. Mol Biol Evol.

[B9] Goldman N, Yang Z (1994). A codon-based model of nucleotide substitution for protein-coding DNA sequences. Mol Biol Evol.

[B10] Muse SV, Gaut BS (1994). A likelihood approach for comparing synonymous and nonsynonymous nucleotide substitution rates, with applications to the chloroplast genome. Mol Biol Evol.

[B11] Kosakovski Pond SL, Frost SD, Muse SV (2005). HyPhy: hypothesis testing using phylogenies. Bioinformatics.

[B12] Anisimova M, Bielawski JP, Yang Z (2001). Accuracy and power of the likelihood ratio test in detecting adaptive molecular evolution. Mol Biol Evol.

[B13] Mantel N (1970). Why step down procedures in variable selection. Technometrics.

[B14] Ben-Bassat M, Krishnaiah PR, Kanal LN (1982). Use of distance measures, information measures and error bounds in feature evaluation. Classification, Pattern Recognition and Reduction of Dimensionality: Handbook of Statistics.

[B15] Miller AJ (1990). Subset selection in regression.

[B16] Akaike H (1973). Information theory and an extension of the maximum likelihood principle. 2nd International Symposium on Information Theory.

[B17] Hurvich CM, Tsai CL (1989). Regression and time series model selection in small samples. Biometrika.

[B18] Posada D, Crandall KA (2001). Selecting the best-fit model of nucleotide substitution. Syst Biol.

[B19] Posada D, Buckley TR (2004). Model selection and model averaging in phylogenetics: advantages of akaike information criterion and bayesian approaches over likelihood ratio tests. Syst Biol.

[B20] Abascal F, Zardoya R, Posada D (2005). ProtTest: Selection of best-fit models of protein evolution. Bioinformatics.

[B21] Self SG, Liang KL (1987). Asymptotic properties of maximum likelihood estimators and likelihood ratio tests under nonstandard conditions. American Statistical Association.

[B22] Yang Z, Swanson WJ, Vacquier VD (2000). Maximum-likelihood analysis of molecular adaptation in abalone sperm lysin reveals variable selective pressures among lineages and sites. Mol Biol Evol.

[B23] Peel M, Donachie W, Shaw A (1988). Temperature-dependent expression of flagella of Listeria monocytogenes studied by electron microscopy, SDS-PAGE and western blotting. J Gen Microbiol.

[B24] Dons L, Eriksson E, Jin Y, Rottenberg ME, Kristensson K, Larsen CN, Bresciani J, Olsen JE (2004). Role of flagellin and the two-component CheA/CheY system of Listeria monocytogenes in host cell invasion and virulence. Infect Immun.

[B25] Hayashi F, Smith KD, Ozinsky A, Hawn TR, Yi EC, Goodlett DR, Eng JK, Akira S, Underhill DM, Aderem A (2001). The innate immune response to bacterial flagellin is mediated by Toll-like receptor 5. Nature.

[B26] The KEGG Database. http://www.genome.ad.jp.

[B27] Schmid KJ, Aquadro CF (2001). The evolutionary analysis of "orphans" from the *Drosophila *genome identifies rapidly diverging and incorrectly annotated genes. Genetics.

[B28] Whelan S, Goldman N (1999). Distributions of statistics used for the comparison of models of sequence evolution in phylogenetics. Mol Biol Evol.

[B29] Kosakovsky Pond SL, Muse SV (2005). Site-to-site variation of synonymous substitution rates. Mol Biol Evol.

[B30] Banfield J, Raftery A (1993). Model-based Gaussian and non-Gaussian clustering. Biometrics.

[B31] Fraley C, Raftery A (1998). How many clusters? Which clustering method? Answers via model-based cluster analysis. The Computer Journal.

[B32] Ren F, Tanaka H, Yang Z (2005). An empirical examination of the utility of codon-substitution models in phylogeny reconstruction. Syst Biol.

[B33] Inagaki Y, Roger AJ Phylogenetic estimation under codon models can be biased by codon usage heterogeneity. Mol Phylogenet Evol.

[B34] Nylander JA, Ronquist F, Huelsenbeck JP, Nieves-Aldrey JL (2004). Bayesian phylogenetic analysis of combined data. Syst Biol.

[B35] Bevan RB, Lang BF, Bryant D (2005). Calculating the evolutionary rates of different genes: a fast, accurate estimator with applications to maximum likelihood phylogenetic analysis. Syst Biol.

[B36] DeLong EF (2004). Microbial population genomics and ecology: the road ahead. Environ Microbiol.

[B37] Doolittle RF (2005). Evolutionary aspects of whole-genome biology. Curr Opin Struct Biol.

[B38] McLachlan GJ, Krishnan T (1997). The EM Algorithm and Extensions.

[B39] Sawa T (1978). Information criteria for discriminating among alternative regression models. Econometrica.

[B40] Sugiura N (1978). Further analysis of the data by Akaike's information criterion and the finite corrections. Communications in Statistics: Theory and Methods.

[B41] Source code and compiled binaries for the described fixed-effect models are available at. http://www.bielawski.info.

[B42] Yang Z (1997). PAML: a program package for phylogenetic analysis by maximum likelihood. Comput Appl Biosci.

[B43] The PAML package. http://abacus.gene.ucl.ac.uk/software/paml.html.

[B44] Lee YH, Ota T, Vacquier VD (1995). Positive selection is a general phenomenon in the evolution of abalone sperm lysin. Mol Biol Evol.

[B45] Moszer I, Glaser P, Danchin A (1995). SubtiList: a relational database for the Bacillus subtilis genome. Microbiology.

[B46] Sequence alignments, phylogenetic trees and gene ontologies for the flagellar system are available at. http://www.bielawski.info.

